# Gynecology

**Published:** 1875-12

**Authors:** 


### I. Gynecology.

1. *Aerotherapy in Chlorosis.* Farre. (*Allgemein. Wien. Medizin. Zeit.*, September, 1875.)

At the altitude of 3,280 feet, mountain air will dispel chlorosis contracted at the level of the sea; but at double the elevation named, anæmia returns. This is due to diminution of oxygen at insufficent elevation—the inverse proportion of oxygen and carbonic acid occurring, not only in the atmosphere, but in the human body. In the atmosphere the gases are mechanically mixed, and the carbonic acid, of greater specific gravity, finds the lowest strata. Its proportion is in inverse ratio to the altitude. Oxygen diminishes but one-seventh where carbonic acid has lost one-half, (Lombard). The low temperature of high altitudes favors condensation, and thus prevents sudden lessening of the proportion of oxygen.

This proportionate difference is more marked in the human body. Atmospheric pressure retards the separation of carbonic acid, which thus accumulates in the organism, and mechanically obstructs the entrance of oxygenated air. The blood saturated with one gas must absorb proportionately less of the other.

In the hygienic treatment of chlorosis then, not the nutrition, but the dwelling place, is the principal factor; not food, but air.

2. *Convulsions due to Morbid Conditions of the Non-Puerperal Uterus.* Graily Hewitt. (*Lancet*, October, 1875.)

With flexion, there is excessive compression of uterine tissue on the concave side of the bend. The thickness of the uterine wall, at the situation where the bend ordinarily occurs, viz., at the junction of the cervix and the body, corresponding to the internal os uteri, is not so great as above that point, but it is considerable. The flexion forces out the blood by a compressing force, which also squeezes the delicate nervous filaments, which everywhere pervade the uterine tissue. It is the compression of the latter, according to the distinguished lecturer, which constitutes the uterine irritation, and produces the convulsions similar to those occurring in the case cited. (Case of acute anteflexion, probably of one year's duration, with frequent convulsive attacks during that period.) Nocturnal exacerbations were explained by the changes induced by the recumbent position. Congestion due to the flexion may account for some symptoms.

Marked uterine flexion exists in all cases of severe repeated hysterical attacks.

Niemeyer is quoted as follows: "flexions, more than any other of the disorders of the uterus, give rise to hysteria." That convulsions do not always result from flexions, is not an argument that they may not thus be induced. The *à priori* argument in favor of this theory is cogent, and the clinical reasons for its support, overwhelming. Special attention is called to the fact, that the painful part of the uterus as discovered by the sound, is always at the seat of the flexion.

3. *Convulsions due to Uterine Polypus.* SPALDING. (*Medical News and Library*, September, 1875.)

A mother of four children had convulsions for two years, the attacks being more frequent at the menstrual period, though rarely absent for a week. One year before their occurrence, there had been uterine hæmorrhage. A polypus, as large as a hen's egg, was removed from its attachment to the anterior wall of the uterus and the external os, by the aid of a wire écraseur. All convulsive symptoms were at once and permanently relieved.

4. *Reduction of a Retroverted Uterus after Six Abortions.* BECK. (*Phila. Med. Times*, October 2, 1875.)

A woman who had aborted five times between the second and fourth month, had retroversion, with pelvic adhesion. The sixth pregnancy was concluded at term, only by careful and skillful treatment, the uterine enlargement occurring principally, if not wholly, from the anterior wall and fundus.

She then aborted at the fourth week from innocent administration of emmenagogues. Ballooning, pessaries, and various modes of reposition were resorted to by Drs. Gregg and Orvis, to relieve the retroversion which was then discovered. Beck, when consulted, found areolar hyperplasia of a large, dense, heavy uterus, situated exactly across the pelvis, the os above the pubic arch, and impinging sharply upon the bladder, the fundus wedged tightly under the promontory of the sacrum, indenting and forcibly pressing upon the rectum. The rectal and urinary symptoms were correspondingly severe. Sims' method of reposition failed, and the protiodide of mercury with ergot, while checking the moderate hæmorrhage, failed to reduce the size of the organ.

Dr. Dills then introduced his hand into the rectum, forcibly broke up the adhesions, and pushed the fundus off from and above the sacral promontory, while Beck made forcible traction upon the os with the tenaculum, and applied heavy pressure from without through the abdominal wall. Under this tremendous force, the fundus returned to its normal position, with a perceptible jump, while the os and cervix properly descended. There was no anæsthesia, and but little pain. The patient walked to her carriage in a few moments; and has required no uterine treatment since.

5. *Extirpation of Uterus by Abdominal Section.* CHADWICK. (*Bost. Med. and Surg. Jour.*, November 4, 1875.)

A patient, fifty years of age, had had one child, and one miscarriage, in early life. There had been more or less metrorrhagia for six years, (three years continuously) and slow but continuous growth of abdominal tumor, on left side. There was emaciation, feebleness, uninterrupted abdominal pain, cephalalgia, vomiting, and hysterical (?) convulsions.

Largest abominal girth was thirty-seven inches, with lateral symmetry. A firm, perfectly round tumor (uterus, with encapsulated fibroid) rose two inches above the navel, and rested on pelvic brim. A body as large as a potato projected from the mass, three inches above and to left of navel. Dr. C. incised the abdomen to the extent of eight inches, and lifted out the uterus, which was free from adhesions. A Wells' clamp was affixed to cervix and broad ligaments, but, to insure safety, a double whip cord was passed through the former and made to include the latter. When the uterus and fibroid tumor were cut away, the left broad ligament slipped from both clamp and noose ; and there was considerable hæmorrhage before the vessels could be secured. The peritoneal cavity was sponged free from blood, the clamp brought to position without much tension, the wound closed with silk sutures, and the whole covered with cotton-wool and adhesive plaster.

The patient rapidly approached convalescence, but died of tetanus on the eighth day ; the internal organs being found, post-mortem, perfectly normal with no trace of blood, serum or lymph in the peritoneal cavity, and no evidences of inflammatory action in any part. The specimen weighed four pounds.

The author justly claims that the fatal result should not depreciate the operation in the eyes of the profession. Péan, with fifteen recoveries in twenty cases, asserts that the danger is no greater than in ovariotomy. The cause of death in this case is common to all surgical operations, and should not be regarded as peculiar to extirpation of the uterus.

6. *Secondary Perineal Traumatism.* MONETTE. (*Am. Practitioner*, October, 1875.)

The perineum of a woman in labor was sustained during expulsion of head, but gave way on emergence of the latter. Rupture was complete to rectum, but did not involve sphincter. The accoucheur then first learned that his patient had suffered a similar accident at her first and prior delivery; the parts having then united by granulation, without the aid of sutures. A similar result occurred in ten days after this delivery, the two cicatrices forming a dense vaginal union.

The reporter infers that union by granulation is possible in all cases, when there is not protracted lochial discharge.

7. *On the Use of Heat in Metrorrhagia.* M. NOËL GUENEAU DE MUSSY. (*Annales de Gynécologie*, July, 1875.)

In many cases of metrorrhagia resisting the action of all classical means, the writer has used, according to the recommendation of Dr. Chapman, applications of water over the sacral region, as hot as the patient could bear them. The water ought to be renewed every two or three hours. In all cases, the hæmorrhage, even the most obstinate, ceased. Dr. Chapman used water at a temperature of 46° C. The writer used the water at a temperature even more elevated.

8. *Early Menstruation.* (*British Med. Jour.*, Sept., 1875.)

A girl aged two years is reported to have menstruated for some time previous to examination. She was perfectly healthy, and the discharge was normal in color, occurring monthly with pain and discomfort.

Another girl, aged eight years, had been subject to fits for two years before examination, the attacks occurring monthly, with intense pubic and sacral pain. The pubis was covered with hair, and a perfect penis, without urethra, was found, over two inches long, capable of erection. Beneath this was a rather large urethra, and a large pendulous vulva. The monthly symptoms were similar to those which accompany the menses.

9. *Ovariotomy at Thirteen.* KOHERLE. (*Gaz. Med. de Strasburg*, Sept. 1, 1875.)

The abdomen enlarged after three months of menstruation. Multilocular cyst of the right ovary was discovered; punctured through a small incision, and removed easily, as there were no adhesions. On the eighth day there were some eructations and vomiting of green ejecta. Pulse, 120; temperature, 103.1°; great thirst and dry skin. Gas was removed from the stomach by the pump, and water injected at intervals of ten, fifteen and twenty hours, with good results. After epistaxis and abscess in each ear, recovery ensued.

10. *Battey's Operation.* YANDELL AND MCCLELLAN. (*American Practitioner*, Oct., 1875.)

This paper is in the dialectic form, and results from an interview had by the authors with the operator.

The latter performs "normal ovariotomy" to rid his patients of "pernicious ovulation," to avail himself of the alterative changes in the nervous system which attend the menopause, and, in doing this, to revolutionize the female economy, and thus eradicate diseases otherwise incurable. The operation is never performed for dysmenorrhœa, amenorrhœa or nyphomania, but solely for relief of disease otherwise incurable.

The patient is placed upon the left side; the cervix seized and drawn under the pubic arch, and the median line of the posterior vaginal cul-de-sac incised with scissors for about one inch and a quarter. The peritoneum, after being rendered accessible, is opened, if there be no hæmorrhage. The ovary is then hooked down into the vagina with the finger, the viscera being depressed, by the hand of an assistant, in the epigastrium. The base of the ovary is temporarily ligated, and the ligature with the ovary removed in ten minutes by the écraseur. Sutures, tents and drainage tubes are discarded ; peritoneal adhesions broken up by the finger. Hæmorrhage is arrested by torsion.

Ten operations have been performed, with two fatal results—one from peritonitis, and another suddenly from a small peritoneal abscess having no outlet. These have been for relief of convulsions, recurring hæmatocele, and recurring pelvic abscess, incipient tuberculosis, ovarian neuralgia and insanity, and intractable dysmenorrhœa with pelvic deposits of "old lymph."

Of the eight cases resulting favorably, five had no untoward symptoms, one suffered from somewhat grave septicæmia which subsided under the peritoneal douche, and two had pelvic peritonitis with abscess and purulent discharge finding vaginal exit.

Sexual desire seems to have been unimpaired after these operations, and, with the exception of the artificially induced menopause and barrenness, no unfavorable sequelæ have been noted.

11. *Drainage of Douglas's Cul-de-sac in Ovariotomy.* SCHROEDER. (*Med. Record*, Sept. 25.)

The author gives the opinions of Peaslee, Sims, Nussbaum and Spiegelberg, who would remove peritoneal accumulations after the operation, by puncture and drainage of Douglas's cul-de-sac, but he cannot concede that the reddish serous exudation is of great etiological importance ; since daily experience teaches that such transudations and exudations into the abdominal cavity do not tend to produce septicæmia and decomposition. Several instances are cited which illustrate this point. Now how does it happen that in one case an exudation, undoubtedly present, occasions no disturbances whatever, does not even perhaps furnish slight symptoms of peritonitis, while, at another time, with a very slight operation, involving scarcely any injury of the peritoneum, there are associated the most violent symptoms of septic peritonitis ? It depends upon whether infection has or has not taken place. In its absence, the exudation is harmless and easily absorbed. Should it occur, the exudation becomes decomposed, or, when there was none before, a violent peritonitis occurs, which rapidly furnishes a decomposing exudation. We must look then not to the accumulating secretion, but to the prevention of the infection which causes it.

Operations, therefore, must be done in healthy places, with absolute cleanliness of hands, linen, clothing, instruments and sponges, and in a disinfected atmosphere. Then drainage is unnecessary and should be omitted, unless, during the operation, pus from a suppurating cyst should enter the abdominal cavity.

When septic peritonitis exists, then, as a therapeutic measure, drainage assumes a very different position. The removal of the exudation is then desirable, but difficult to accomplish. The cul-de-sac is easily punctured only when an exudation is enclosed in it. It is precisely in these cases, however, that the evacuation is not absolutely demanded, for, the exudation being encapsulated, it is rendered harmless and does not lead to absorption or perforation. If the exudation, however, is general and free, the cul-de-sac does not bulge. In puerperal peritonitis (the cervix of the anteverted uterus lying close to the rectum) there are two alternatives: abdominal incision, which is unsatisfactory as an aid to washing out the cavity, and laparotomy, with perforation and drainage from within. This may yet become the treatment for septic peritonitis, though it requires great confidence to undertake it, in a woman suffering from general peritonitis.

12. *Rupture of Ovarian Cysts into the Peritoneum.* NEPVEN. (*Annales de Gynécologie*, July, 1875.)

There were 63 fatal cases of the 127 collected by the author; complete cure in 43; reformation of cyst in 21.

In 49 cases the contents were designated, and of these, 4 were cured; all were serous effusions. In 19 cases there was caseous or liquid pus; of the rest, 7 cysts were serous, 4 cancerous, 2 hydatid, 2 dermoid, and 7 gelatinous.

All were treated by immediate puncture of the abdomen, according to Spencer Wells's method in ovariotomy.

The author has collected 27 cases also of rupture into neighboring organs; 6 into the bladder, 2 into the vagina, 1 into the uterus, and 7 externally through the abdominal wall.

